# Monkeypox outbreak: Should Bangladesh be worried?

**DOI:** 10.1016/j.amsu.2022.104685

**Published:** 2022-09-15

**Authors:** Abhigan Babu Shrestha, Fahmida Hoque Rimti, S.M. Fuad Hasan, Manjil Aryal, Mst Arefin Naher, Sajina Shrestha, A.M. Khairul Islam

**Affiliations:** M Abdur Rahim Medical College, Dinajpur, Bangladesh; Chittagong Medical College, Chittagong, Bangladesh; M Abdur Rahim Medical College, Dinajpur, Bangladesh; KIST Medical College, Imadol, Patan, Nepal; International Centre for Diarrhoeal Disease Research, Bangladesh (ICDDR,B), Mohakali, Dhaka, Bangladesh

Dear Editor,

Monkeypox virus (MPX) is a zoonotic disease caused by a double DNA virus, a member of the Orthopox virus. Although the MPX has circulated for decades in regions where it has traditionally been endemic, research into MPX has been remission and inefficient. More than 16000 cases and 5 deaths have been reported in 75 countries since May 2022 persuading the World Health Organization [WHO] to declare the MPX outbreak as a public health emergency of international concern [1]. As of 10 August, 162 MPX cases were found in Asia. Of these, nine cases were reported from India. Since Bangladesh shares a larger common border with India there is no reason for Bangladesh to feel relaxed reflecting the situation during the COVID-19 pandemic [2] again, UAE also confirms its monkeypox cases among the population. According to the Bureau of Manpower, Employment and Training (BMET), the UAE has recruited 2.37 million Bangladeshi workers this September [3]. The first case in India was a migrant from UAE. Keeping this in mind, Bangladesh also has a large number of a migrant in UAE making it more vulnerable. This is especially concerning as the knowledge level of monkeypox among the general population of Bangladesh is quite poor [14].

On the 7th and 9th of June, three suspected cases were found in Bangladesh which caught the eye of the public and health policy makers [[4], [5], [6]]. The first case was suspected at the airport of a 32-year-old Turkish citizen while screening. He was then taken to the Infectious Diseases Hospital in Dhaka's Mohakhali. However, on 9th June the case was reported negative. And, the second time, two cases were seen, one was isolated in Chuadanga in a 60-year-old woman after local doctors detected symptoms of MPX. On the same day, a 42 years old man returning from India through Benapole Border Crossing was sent to Jashore Hospital after showing pox-like features.

The disease presentation is similar to smallpox, such as fever accompanied by headache, rash, back pain, malaise, fatigue, lymphadenopathy, and centrifugally distributed vesicopapular rash. The incubation period of the virus ranges from 10 to 14 days [7]. However, definitive treatment hasn't been established for treating this new global threat. To date, vaccines seem to be a plausible answer for preventing their spread. As of now ACAM2000 and JYNNEOS are the only two vaccines recommended for preexposure prophylaxis [8]. WHO recommends a Ring Vaccination strategy for a high-risk group of MPVX exposure (similar to Ebola) [9] This concept is highly advisable in countries with a shortage of stockpiles. Lower middle incomes countries like Bangladesh can take maximum benefit from applying this. The high-risk group is divided into pre and post-exposure; pre-exposure includes those with occupational hazards like healthcare professionals, laboratory personnel, and outbreak response team member [9] Bisexual, Gay, and other men who have sex with men (GBMSM) are also considered a preexposure high-risk group. Post-exposure prophylaxis is recommended for contacts of confirmed cases. Antivirals (like tecovirimat, brincidofovir, cidofoviretc.) [10], and vaccinia immune globulin intravenous (VIGIV) [11] are also available. They are considered either in severe diseases or immunocompromised, pregnant and breastfeeding mothers, and lesions complicated or occurring near the mouth, eyes, and genitals [8].

Amidst the scarcity of sufficient healthcare resources in Bangladesh, doctors should be promptly discerning the presenting signs and symptoms of the disease rather than solely relying on symptomatic treatment for addressing a possible outbreak in the country. In addition to these, hospitals ought to have functional isolation units to readily admit suspected patients which in turn should help in containing the outbreak in a similar manner taken during COVID-19 preparedness. A constructive and efficient surveillance and monitoring system for tracking cases and analyzing them should be implemented. The persons who are in close contact with the cases should also be effectively traced and monitored. In cases of contagious diseases like MPX, accurately informing mass people through public sessions can significantly help in reducing the chance of an outbreak. Screening tests should be mandated at the ports of entry to the country for the people traveling from the places where the number of MPX cases is increasing. Keeping this in mind, on 22nd May, the Directorate General of Health Services (DGHS) issued a warning at every possible entry port including all air, land, and sea ports. Suspected cases were ordered to be sent immediately to the hospital and isolated. [12] with this Bangladesh becomes the first nation to bar shore passes to MPX fear. As the smallpox vaccine may protect against monkeypox or may decrease the severity of the disease, the Bangladesh government should build a solid infrastructure for immunizing people on a priority basis. Meanwhile, the WHO has issued recommendations for specific communities namely gay, bisexual and other men who have sex with men (MSM) due to the asymmetric number of cases among this population, although MPX is not a sexually transmitted disease. Stigmatizing a disease like this could become a real hindrance in tackling a possible outbreak, especially in countries like Bangladesh considering its socioeconomic condition. Moreover, the social stigma associated with this re-emerging disease makes people hesitate to refer to clinics and hospitals to keep it secret to prevent being isolated from society. Instead, the fact that anyone can be affected with MPX through broken skin, respiratory droplets, or the mucous membranes (eyes, nose, or mouth) of an infected person should be established. Even soon if cases are spotted, it will be a great tackle to control the potential outbreak due to lack of contact tracing system, self-medication practice [13], and lack of timely diagnosis of MPX.

Bangladesh has faced a severe downfall in its health sector due to a massive hit of previous and ongoing variants of COVID-19, in addition, global inflation has added another burden to the cost of living in this lower middle-income country. Hence, a new outbreak could lead to a massive health crisis. Moreover, Bangladesh is a vulnerable country to any contagious diseases because of its geographical and ecological condition, population, and transborder communication. To our concern, enough preparedness hasn't been to furnish the core health sectors. Hence, it is high time that government and non-governmental allies take control of the spark before the fire breaks out.

## Ethical approval

N/A.

## Data availability statement

Data sharing is not applicable to this article as no datasets were generated or analyzed during the current study.

## Sources of funding

This research did not receive any specific grant from funding agencies in the public, commercial, or not-for-profit sectors.

## Author contribution

All authors have participated in writing and reviewing the manuscript, All authors have approved the final draft of the manuscript.

## Registration of research studies

Not applicable.

## Consent

NA.

## Guarantor

Abhigan babu shrestha.

## Declaration of competing interest

All authors declare no conflict of interest.

## References

[1] WHO Director-General declares monkeypox outbreak a public health emergency of international concern - PAHO/WHO | Pan American Health Organization. https://www.paho.org/en/news/23-7-2022-who-director-general-declares-monkeypox-outbreak-public-health-emergency.

[2] (2022). 2022 Monkeypox Outbreak in Asia.

[3] T.F. Express Dhaka to urge UAE for providing workers' job visa, DFQF access, the Financial Express. https://thefinancialexpress.com.bd/trade/dhaka-to-urge-uae-for-providing-workers-job-visa-dfqf-access-1636426674.

[4] জনকণ্ঠদৈনিক, মাঙ্কিপক্স নেগেটিভ সেই তুর্কি নাগরিকের, দৈনিক জনকণ্ঠ || Daily Janakantha. https://www.dailyjanakantha.com/national/news/654010.

[5] (2022). Health Ministry Dismisses Monkeypox Reports.

[6] UNB B. (2022). https://www.thedailystar.net/health/disease/news/india-returnee-monkeypox-symptoms-sent-jashore-hospital-3044326.

[7] Brown K., Leggat P.A. (2016). Human monkeypox: current state of knowledge and implications for the future. Trop Med Infect Dis.

[8] Rizk J.G., Lippi G., Henry B.M., Forthal D.N., Rizk Y. (2022). Prevention and treatment of monkeypox. Drugs.

[9] (14 June 2022). Vaccines and Immunization for Monkeypox: Interim Guidance.

[10] Clinical features and management of human monkeypox: a retrospective observational study in the UK - the Lancet Infectious Diseases. https://www.thelancet.com/journals/laninf/article/PIIS1473-3099(22)00228-6/fulltext.

[11] Vaccinia immune globulin: current policies, preparedness, and product safety and efficacy - international Journal of Infectious Diseases. https://www.ijidonline.com/article/S1201-9712(06)00040-3/fulltext.

[12] (2022). Monkeypox: Govt Puts Ports on Alert, the Business Standard.

[13] Shrestha A.B., Aryal M., Magar J.R., Shrestha S., Hossainy L., Rimti F.H. (2022). The scenario of self-medication practices during the covid-19 pandemic; a systematic review. Annals of Medicine and Surgery.

[14] Nath, Assessment of knowledge on human monkeypox virus among general population in Bangladesh: a nationwide cross-sectional study, Medrxiv, doi:10.1101/2022.08.31.22279445, In press.

